# Clinical Correlation of Oblique Magnetic Resonance Imaging for Cervical Foraminal Stenosis

**DOI:** 10.7759/cureus.78130

**Published:** 2025-01-28

**Authors:** Juan I Straface, Carla García-Ramos, Ruben Conde-Espinoza, Mayra C Osuna-Espinoza, Jose L Barragan-Hermosillo, Jorge L Acosta-Cortez, Barón Zárate-Kalfópulos, Alejandro Antonio Reyes-Sanchez

**Affiliations:** 1 Spine Surgery, National Institute of Rehabilitation Luis Guillermo Ibarra Ibarra, Mexico City, MEX; 2 Radiology, National Institute of Rehabilitation Luis Guillermo Ibarra Ibarra, Mexico City, MEX; 3 Orthopaedic Surgery, National Institute of Rehabilitation Luis Guillermo Ibarra Ibarra, Mexico City, MEX

**Keywords:** cervical foraminal stenosis, cervical spine, clinical correlation, oblique mri, park classification

## Abstract

Introduction

The diagnosis of cervical foraminal stenosis is primarily clinical since imaging methods do not allow direct visualization of the foramen, limiting the usefulness of classic studies such as simple magnetic resonance imaging. The objective of the study was to correlate the clinical symptoms of patients with cervical foraminal stenosis with the images obtained from oblique cervical spine magnetic resonance imaging.

Materials and methods

A pilot study included patients diagnosed with cervical foraminal stenosis. Clinical evaluation and disability scales [Neck Disability Index (NDI), modified Japanese Orthopedic Association Scale (mJOA)], quality of life [Short Form-36 (SF-36)], and pain [visual analog scale (VAS)] were applied. Subsequently, oblique magnetic resonance imaging at 45° was performed with direct visualization of the left and right foramina, and stenosis grades were classified using the Park scale. Descriptive statistics and correlations between clinical scales and stenosis grades were performed.

Results

The sample consisted of 30 patients who met the inclusion criteria, with an average age of 56.37 ± 15.81 years. 50% (15/30) were female. The SF-36 mental component summary (MCS) scale reported an average of 40.23 ± 13.29 (range 15-70), SF-36 physical component summary (PCS) 40.23 ± 12.77 (range 19-65), NDI 28.90 ± 16.60 (min 3, max 80), mJOA 10.70 ± 3.04 (range 4-17). The correlation between the Park compression degree and age was significant (r = 0.735, p = 0.0001), indicating greater stenosis with increasing age. A significant correlation was also found between the mJOA clinical scale and Park compression degree (r = 0.41, p = 0.022).

Conclusion

A significant correlation was found between age and the degree of foraminal stenosis, as well as between foraminal stenosis according to the Park scale and the mJOA scale.

## Introduction

Cervical foraminal stenosis is a condition characterized by the narrowing of the foramina in the cervical spine. This narrowing can compress the spinal nerves, leading to symptoms such as neck pain, radiating arm pain, numbness, and muscle weakness. Causes include degenerative changes in the spine, such as herniated discs, bone spurs, or thickened ligaments. Diagnosis is primarily clinical, as imaging methods fail to directly visualize the foramina [1-5]. Current standard projections, such as axial, sagittal, and coronal, do not clearly show nerve root anomalies and entrapment in the foraminal area [5]. However, oblique MRI at 45° exposes the cervical foramen with all its borders in a single image [6-9]. Park proposes a system for diagnosing and classifying cervical neuroforaminal stenosis using this projection [6, 7], which has demonstrated sensitivity, specificity, and accuracy of 96.7%, 95.0%, and 96.0%, respectively [7, 10]. Despite these results, no studies have correlated clinical data with this classification scale, prompting the objective of this study to correlate clinical symptoms of patients with cervical foraminal stenosis and the images obtained from oblique MRI [10-14], which could help standardize the oblique vue in all cervical MRIs in the context of cervical pain for more precise diagnosis.

## Materials and methods

A pilot study was conducted, approved by the institutional committee (number 2/21), and the first 30 patients diagnosed with cervical foraminal stenosis found in an outpatient clinic in September 2024 were included. Clinical scales [visual analog scale (VAS), Short Form - 36 (SF-36), Neck Disability Index (NDI), modified Japanese Orthopedic Association Scale (mJOA)] and cervical MRI with oblique cuts were reviewed, and written informed consent was obtained, and saved in patient files.

Patients with previous neck surgery, brachial plexus pathology, or peripheric nerve compression were excluded from the study.

Descriptive statistics were used for quantitative variables, including measures of central tendency (mean, median, mode) and dispersion (standard deviation, minimum, maximum, and range). Qualitative variables were assessed as percentages. The Shapiro-Wilk test was applied to evaluate normality, we considered a significant difference with a p < 0.05. 

Pearson's correlation to investigate relationships between clinical scales and the degree of degeneration by level according to the Park scale. Inter- and intra-observer agreement by two spine surgeons with over 10 years of experience was evaluated using Cohen's kappa, yielding a value of 0.78. A p-value of < 0.05 was considered statistically significant. Data was analyzed using SPSS (IBM Corp. Released 2011. IBM SPSS Statistics for Windows, Version 20.0. Armonk, NY: IBM Corp), with graphs prepared in Excel. The database is securely stored in the spine surgery research department.

Digitalized and anonymized MRI images were presented in two randomized sessions, with a two-week interval, for classification by two different MRI experts, each with more than 10 years of experience (k=0.82), thereby ensuring blinding to reduce bias. The images were then classified according to the Park grading scale, which has demonstrated moderate to relatively high correlations between grade and neurological manifestations based on cervical level (R = 0.570-0.715; all p < 0.05) [11].

Oblique magnetic resonance imaging at 45° was performed on both sides. Patients with prior cervical spine surgeries, brachial plexus pathologies, and peripheral nerve compressions were excluded. MRI was for each patient using the same protocol on a 3-T magnet (Intera, Philips Healthcare) using a SYN head coil (Philips Healthcare) and fast spin-echo imaging. The MRI parameters used were the following: axial T2-weighted turbo spin-echo [repetition time (TR)/ echo time (TE), 2500/100; field of view (FOV), 17 cm; matrix, 315 × 250; slice thickness, 3 mm; signal average, 1; interslice gap, 0.3 mm]; sagittal T2-weighted spin-echo (TR/TE, 2500/100; FOV, 24 cm; matrix, 360 × 280; slice thickness, 4 mm; signal average, 3; interslice gap, 0 mm); axial T1-weighted spin-echo (TR/TE, 700/10; FOV, 17 cm; matrix, 315 × 250; slice thickness, 3 mm; signal average, 1; interslice gap, 0.3 mm); and sagittal T1-weighted spin-echo (TR/TE, 500/10; FOV, 24 cm; matrix, 360 × 280; slice thickness, 4 mm; signal average, 3; interslice gap, 0 mm). Oblique sagittal images were obtained by identifying the medial edge of the neural foramen, then, seven images were obtained progressing laterally through the foramen with a projection angle of 45° on both sides of the neck. The angled projections were obtained with the following parameters: TR range/TE, 1400-2500/100; FOV, 24 cm; matrix, 360 × 280; slice thickness, 3 mm; interslice gap 0 mm; and signal average, 3 [6, 12-16].

The oblique T2 sequence MRI was evaluated by two independent expert observers (inter-observer variability κ = 0.84) using digital imaging and communications in medicine (DICOM) system images. The left and right diameters of cervical levels from C2-C3 to C7-T1 were measured according to the Park scale [7]. Clinical evaluations were conducted using functional scales, including the SF-36 physical component summary (PCS) for the physical aspect, the SF-36 mental component summary (MCS) for the mental aspect [17], the Neck Disability Index (NDI) [18], and the modified Japanese Orthopaedic Association scale (mJOA) [19].

## Results

**Figure 1:**
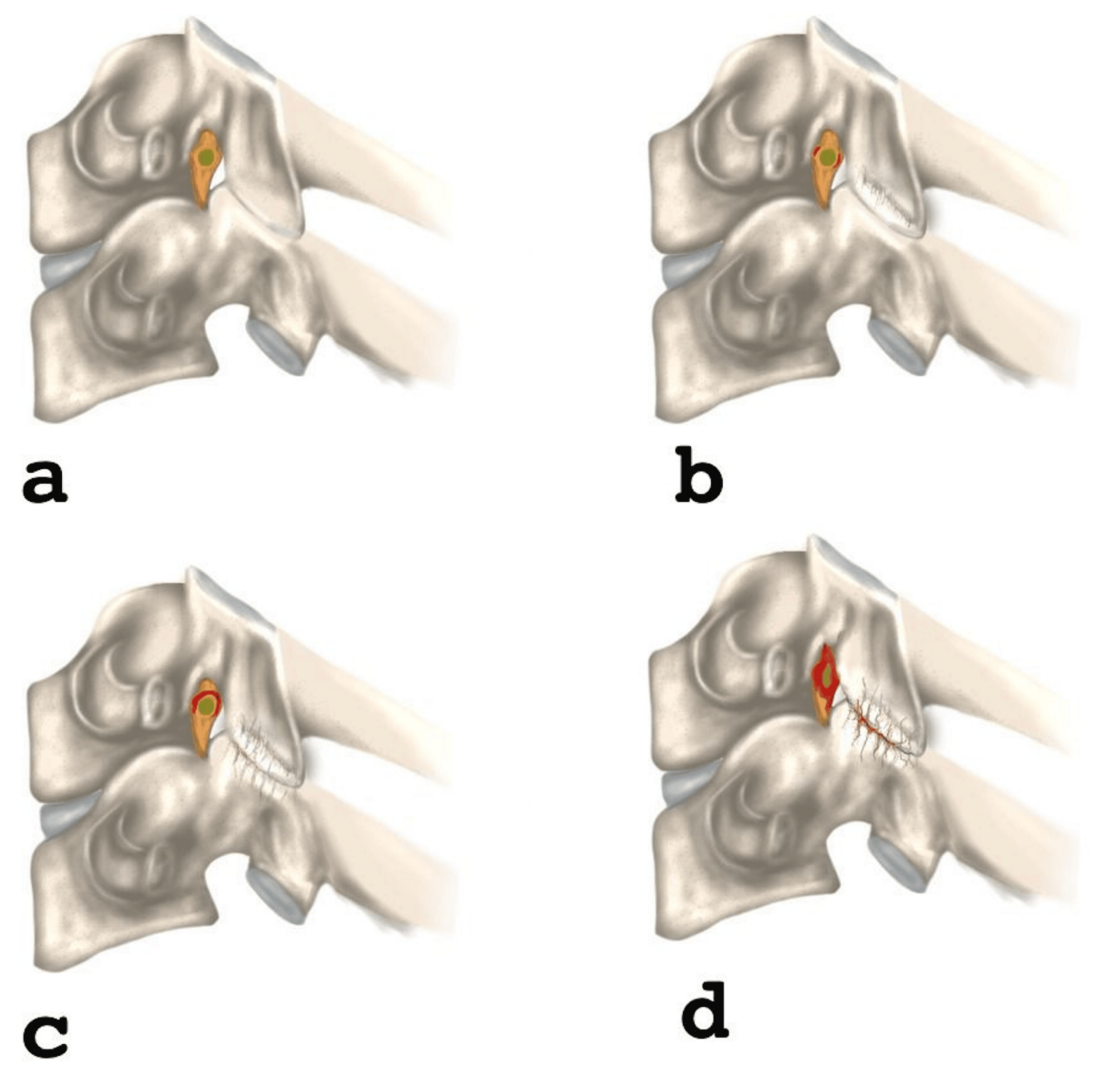
Schematic illustrations of Park grading system for cervical foraminal stenosis (a) Grade 0 shows no significant stenosis and preserved perineural fat without obliteration. (b) Grade 1 shows to mild stenosis with less than 50% perineural fat obliteration around the nerve root and no morphological changes in the nerve root. (c) Grade 2 shows moderate stenosis with more than 50% perineural fat obliteration but still no morphological changes in the nerve root. (d) Grade 3 shows severe stenosis characterized by complete obliteration of perineural fat, nerve root collapse, and morphological changes in the nerve root.

**Figure 2:**
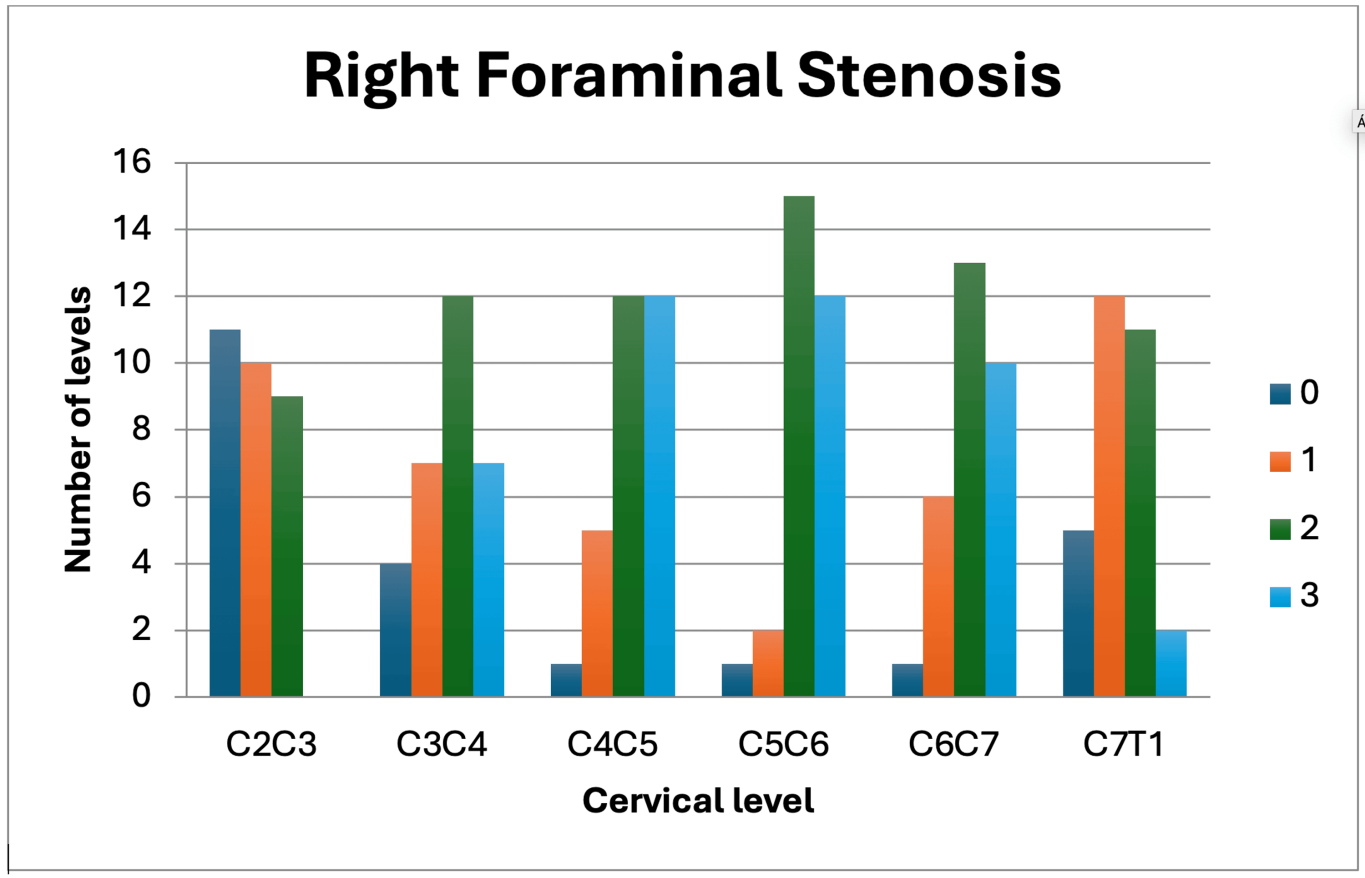
Foraminal stenosis of the right Park Classification according to the studied levels on the right side of the spine.

**Figure 3:**
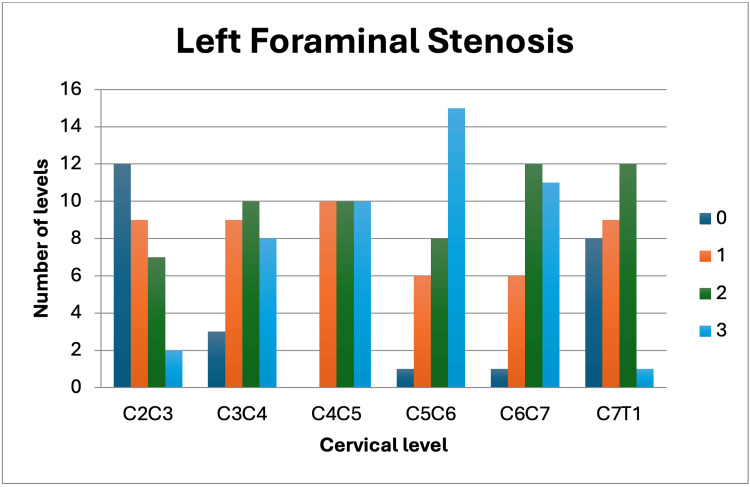
Foraminal stenosis of the left Park Classification according to the studied levels on the left side of the spine.

The sample included 30 patients diagnosed with cervical foraminal stenosis, with an average age of 56.37 ± 15.81 years, of which 50% (15/30) were female. Patient demographics, along with average scores for SF-36 MCS (40.23 ± 13.29), SF-36 PCS (40.23 ± 12.77), NDI (28.90 ± 16.60 points), and mJOA (10.70 ± 3.04 points), are presented in Table 1. Park's classification (Figure 1) by level is detailed in Table 2, showing a total of 360 levels analyzed. Figure 2 shows the Park's classification on the right side and Figure 3 shows the left side. Significant correlations were found between the degree of Park compression and age (r = 0.735, p = 0.0001), as well as between the mJOA clinical scale and Park compression degree (r = 0.41, p = 0.022). However, no associations were observed between other clinical scales, gender, and foraminal compression degree according to the Park classification. A group of nine patients presented a higher degree of degenerated discs according to the Park scale, and a correlation was performed using the clinical scales without obtaining significant data. However, this group presented a strong correlation with age (r = 0.50, p = 0.015).

**Table 1:** Patient demographics and scales M: means media; SD: standard deviation; SF: Short Form; PCS: physical component summary; MCS: mental component summary; NDI: Neck Disability Index; mJOA: modified Japanese Orthopaedic Association scale; *p=<0.05.

Parameter	Scale	Results	Sig.
N=30			
Age, M±SD	Years	56.37±15.81	0.607
Sex, n(%)	Female	15(50%)	0.002*
	Male	15(50%)	
Clinical scales			
SF-36, M±SD	MCS	40.23±13.29	0.598
	PCS	40.23±12.77	0.471
NDI, M±SD	pts	28.90±16.60	0.56
mJOA, M±SD	pts	10.70±3.04	0.649

**Table 2:** Park classification by studies level

	Right cervical foramen						Left cervical foramen						
Park	C2C3	C3C4	C4C5	C5C6	C6C7	C7T1	C2C3	C3C4	C4C5	C5C6	C6C7	C7T1	Total
0	11	4	1	1	1	5	12	3	0	1	1	8	48
1	10	7	5	2	6	12	9	9	10	6	6	9	91
2	9	12	12	15	13	11	7	10	10	8	12	12	131
3	0	7	12	12	10	2	2	8	10	15	11	1	90
Total	30	30	30	30	30	30	30	30	30	30	30	30	360

## Discussion

Cervical spine MRI is the gold standard for diagnosing foraminal stenosis, confirming the diagnosis, and locating the affected level and the region of nerve root compression. However, imaging has limitations as nerve root pathways are not in standard orthogonal planes, and nerve root diameter is 2-3 mm. Routine T2-weighted axial and sagittal MRI sequences are not the most objective method for determining foraminal stenosis. In clinical settings, there is no objective evaluation method for describing foraminal stenosis, leading to subjective terms like "mild," "moderate," and "severe" [20].

Other classification systems such as the Kang MRI [21] grading system provide an alternative method for assessing cervical canal stenosis, primarily using axial and sagittal MRI sequences to correlate imaging findings with clinical symptoms. While this system has demonstrated clinical utility, it does not incorporate oblique sagittal imaging, a key feature of the Park grading system used in this study. The Park scale allows for a detailed evaluation of neuroforaminal narrowing, offering a more precise correlation with functional outcomes such as radicular pain and disability. Incorporating insights from both grading systems could enhance the overall diagnostic approach, combining Kang's broader scope of canal assessments with the focused neuroforaminal analysis provided by Park [6]. This complementary use underscores the importance of advancing imaging techniques to improve the evaluation and management of cervical spine pathologies.

Park et al. [6, 10] used clinical correlation of oblique MRI, comparing cervical foraminal stenosis according to the Park scale at three cervical levels (C4-C5, C5-C6, C6-C7) with clinical manifestations such as paresthesia, limb weakness, numbness, and/or radicular pain, along with clinical signs like Lhermitte and Spurling's sign, reduced osteotendinous reflexes, and/or electromyography (EMG) changes. More than one clinical neurological manifestation combined with more than one clinical neurological sign was considered to correspond with cervical foraminal stenosis. A clinical correlation between grade and neurologic manifestations was found in R = 0.565 and R = 0.675.

Lee et al. [11] studied 188 patients, evaluating only three cervical neurological levels (C4-C5, C5-C6, C6-C7), considering at least one clinical manifestation and one combined clinical neurological sign as a positive neurological manifestation (PNM). No electrodiagnostic studies were performed on any patient. They found high correlations between grade and neurologic manifestations based on cervical level (R = 0.570-0.715) (all p < 0.05) without specifying the clinical scales they used to measure. Meacock et al. discussed methods for classifying cervical foraminal stenosis however, they did not correlate with clinical findings. Kim et al. and Park et al. are the most accepted systems, but the clinical application of these scores is limited by non-standard images, and validation is limited against clinical symptoms and surgical outcome data [10, 12]. Our study reported R = 0.41 for the Park grade, being less than other studies, probably due to the higher age associated with more degenerative changes.

This study doubled the comparison with six cervical levels and evaluated only 30 patients. To perform clinical correlation, we used three validated functional scales (SF-36, NDI, mJOA), with only mJOA showing a statistically significant correlation with cervical foraminal stenosis according to the Park scale, where higher scores indicate better functional status. These results are similar to our own. The superiority of mJOA over the NDI is probably due to the nature of both scales, while the NDI emphasizes quality of life, mJOA emphasizes neck symptoms. As most outpatients were grade 1 or 2, the quality of life in these patients tends not to be as affected. A larger study would likely show better validity in patients with more compression. Age also showed a statistically significant correlation with stenosis degree, aligning with chronological joint changes.

Fine-cut oblique images from 3D MRI data sets can produce more consistent, better clinical correlation, improved outcomes, and surgical better decision-making [22, 23]. Barnaure et al. [16] demonstrated the efficacy of MRI in evaluating cervical foraminal stenosis by comparing 3D T2 SPACE oblique sagittal cuts and 2D T2 TSE, showing T2W oblique sequences are more accurate in the assessment of cervical foramina than conventional sagittal and axial sequences, potentially altering surgical decisions. Differences in foraminal classification were not significant for most levels or showed minor clinically acceptable differences. Hersh et al. [23] proposed new alternatives for studying cervical foraminal stenosis using enlarged cuts in CT to better visualize foramina compared to traditional sagittal and axial CT cuts, being reproducible in the absence of oblique MRI, reducing study time, and evaluating foramina bilaterally unlike oblique MRI in cervical foraminal stenosis. Despite demonstrating the effectiveness of oblique MRI in evaluating cervical foraminal stenosis and its impact on staging and therapeutic decisions, no indexed studies have compared functional clinical scales with cervical foraminal stenosis degree using oblique MRI and Park scale. Our results suggest using the mJOA scale for clinical evaluation as it showed a statistically significant clinical correlation for cervical foraminal stenosis.

Our study has some limitations. The sample size is small, which may limit the generalizability of the findings to a broader population. Additionally, it is not possible to definitively determine whether the reported pain is directly conditioned by foraminal stenosis, as the clinical scales are utilized to measure overall disability rather than isolating specific pain sources. Future studies with larger sample sizes are needed to better understand this relationship.

One of the significant contributions of this study is the inclusion of cervical levels that had not been correlated with clinical symptoms in prior research. By employing the Park grading system with oblique MRI, we demonstrated that these previously unexamined levels could also be effectively analyzed. This expands the utility of the grading system and oblique MRI in clinical practice. Future studies could aim to include a larger cohort of patients and incorporate correlations with other diagnostic tools, such as electromyography, to validate and enhance the diagnostic value of the Park grading system further. Additionally, longitudinal studies could assess how these correlations evolve over time and influence treatment outcomes.

## Conclusions

Oblique magnetic resonance imaging and the Park grading system provide a valuable framework for evaluating cervical foraminal stenosis by enabling detailed visualization and correlation with clinical symptoms. This study highlights the significant relationship between foraminal stenosis severity and functional outcomes, as demonstrated by the mJOA scale. Incorporating advanced imaging techniques like oblique MRI into routine practice can improve diagnostic accuracy and guide clinical decision-making, ultimately enhancing patient management. Future research should focus on larger cohorts and longitudinal analyses to validate these findings and refine the application of such imaging modalities.
